# Evaluation of Injuries Caused by Sharp Objects in Different Suicide Cases

**DOI:** 10.15388/Amed.2026.33.1.11

**Published:** 2026-07-22

**Authors:** Jekaterina Strelčenko, Sigitas Chmieliauskas, Sigitas Laima, Diana Vasiljevaitė, Jurgita Stasiūnienė, Paulius Petreikis

**Affiliations:** 1 Vilnius University, Faculty of Medicine, Vilnius, Lithuania; 2 Department of Pathology, Forensic Medicine, Institute of Biomedical Sciences of the Faculty of Medicine of Vilnius University, Vilnius, Lithuania; 3 Department of Anatomy, Histology and Anthropology, Institute of Biomedical Sciences, Faculty of Medicine, Vilnius University, Vilnius, Lithuania

**Keywords:** suicide, sharp object, stab wound, cut injuries, forensic pathology, savižudybė, aštrūs daiktai, durtiniai sužalojimai, pjautiniai sužalojimai, teismo medicina

## Abstract

**Background:**

Suicide, defined as the act of taking one’s own life, is a common cause of death worldwide, with more than 720,000 people dying by suicide each year. However, suicide by sharp objects is relatively rare, accounting for only a small percentage of all cases. In some cases, self-inflicted injuries may be mistaken for other causes, such as assault or accidental trauma. The mortality rate from self-inflicted injuries with sharp objects is low, and most such suicide attempts do not lead to death. The low fatality rate associated with self-inflicted sharp force injuries may prompt the use of an additional method, which characterizes a complex suicide. Therefore, in this article, we will analyze the injuries caused by sharp objects in different suicide cases.

**Materials and methods:**

In this retrospective study, we analyzed injuries caused by sharp objects in complex suicides with the objective to identify correlations with the suicide methods used. From 2019 to 2024, 98 nonconsecutive, impersonalized suicide autopsy cases (71 men and 27 women) involving self-inflicted cuts or stab wounds were selected from data provided by the State Forensic Medicine Service of Lithuania. All victims underwent full autopsies, including toxicological testing for alcohol and drugs. A literature search was conducted in the *PubMed* database by using relevant keywords. The analysis focused primarily on studies from the last 10 years but also included older studies developing strong arguments.

**Results:**

The mean age of the deceased in the study sample was 45.1 ± 17.9 years, with no difference between the sexes. In more than half of the cases (n = 52), suicides occurred while sober. Among alcohol-intoxicated individuals (n = 46), the mean blood alcohol concentration (BAC) was 1.96‰ ± 1.06. The most frequent main cause of death after unsuccessful self-inflicted injuries with sharp objects was hanging, followed by exsanguination and drug poisoning. In cases of hanging, the mean age was 45 ± 15, 60% were intoxicated (n = 32), with a mean BAC of 2.04‰ ± 1.08. A median of 13 trial cuts was found, most commonly on the forearms. When the primary cause of death was exsanguination due to cut/stab wounds, 41% were intoxicated with alcohol (mean BAC was 1.92‰ ± 0.76). In this group, 59% (n = 10) had cut neck wounds affecting major vessels, 23% (n = 4) had stabbed wounds to the chest with damage to the heart and main blood vessels, 18% had cuts to the upper limbs involving arteries. The mean age of individuals varied by injury location, notably, neck at 61 ± 20 years, chest at 39 ± 12 years, upper limbs at 71 ± 9 years. In all cases, no clothes damage was observed. A mean of 8 ± 4 (median 8) superficial neck cuts were found near the main wound in fatal neck injuries, 5 ± 2 (median 6) in the lethal chest injuries, and 6 ± 1 (median 6) in the death-causing upper limb injuries. The number of trial wounds on the neck, chest, and arms did not differ significantly (*p* >0.05), but was significantly higher in the hanging group (*p* <0.05). Concerning the third most frequent cause of death, a median of 16 trial cuts was found, of which, 42% on both forearms. There was no statistically significant difference in the mean age between cases of drugs poisoning and hanging (*p* >0.05), while exsanguination cases showed significant difference (*p* <0.05). A negative correlation was observed between the age of the entire study group and the number of trial wounds.

**Conclusions:**

Suicides by sharp objects are a relatively rare phenomenon; they most commonly involve multiple superficial trial injuries alongside a fatal stab or incised wound. Suicides with sharp objects are most common among middle-aged men, and, in more than half of the cases, the individuals were sober at the time of the event. The majority of trial cuts were observed on the upper limbs, especially in cases of hanging. In all instances, no damage to clothing was observed. Recognition of injury patterns and locations, along with the absence of defensive wounds and clothing damage, could be essential for accurate forensic interpretation, particularly in complex and ambiguous cases.

## Abstract (it)

**Įvadas:**

Savižudybė – savo gyvybės atėmimas, yra dažna mirties priežastis visame pasaulyje. Kasmet daugiau nei 720 000 žmonių miršta nusižudydami. Tačiau savižudybės, padarytos pjaunančių ir duriančių savybių daiktu, yra retesnės ir sudaro tik kelis procentus visų savižudybių. Kartais tokie savęs sužalojimai gali būti supainiojami su nužudymu ar nelaimingu atsitikimu. Mirtingumas nuo savęs sužalojimo aštriais daiktais yra santykinai mažas, todėl dalis savižudybių bandymų nesibaigia mirtimi. Mažas mirtingumas, susijęs su savęs sužalojimu aštriais daiktais, gali paskatinti naudoti papildomą metodą, kas apibūdina kompleksinę savižudybę. Šiame straipsnyje analizuosime sužalojimus, padarytus aštriais daiktais įvairių savižudybių atvejais.

**Medžiagos ir metodai:**

Šiame retrospektyviniame tyrime analizuojami savižudybių metu padaryti pjautiniai ir durtiniai sužalojimai, siekiant nustatyti sąsajas su skirtingais savižudybės būdais. Nuo 2019 iki 2024 metų iš Lietuvos valstybinės teismo medicinos tarnybos pateiktų nuasmenintų duomenų atrinktos 98 autopsijos (71 vyro, 27 moterų), kai mirties priežastis savižudybė ir buvo durtinių ir pjautinių sužalojimų. Visų mirusiųjų buvo atliktos išsamios autopsijos su toksikologiniais – etilo alkoholio, medikamentų bei kitų toksinių, narkotinių medžiagų tyrimais. Mokslinės literatūros buvo ieškoma „PubMed“ duomenų bazėje, naudojant atitinkamus raktinius žodžius. Analizė sutelkta į pastarųjų 10 metų tyrimus, tačiau įtraukti ir senesni tyrimai su svariais argumentais.

**Rezultatai:**

Tiriamosios imties mirusiųjų amžiaus vidurkis 45,1±17,9 metai. Statistiškai reikšmingo amžiaus skirtumo tarp lyčių nenustatyta. Daugiau nei pusę atvejų (n = 52) sudaro savižudybės esant blaiviems. Apsvaigusiųjų (n = 46) etilo alkoholio koncentracijos vidurkis kraujyje (AKK) 1,96‰±1,06. Dažniausia pagrindinė mirties priežastis po bandomųjų savęs žalojimų aštriais daiktais yra pasikorimas, nukraujavimas ir apsinuodijimas medikamentais. Pasikorusiųjų amžiaus vidurkis 45±15 metų, 60 % buvo apsvaigę nuo etanolio, kai AKK vidurkis 2,04‰±1,08. Pasikorimo atvejais nustatyta 13 bandomųjų pjūvių skaičiaus mediana, dažniausiai dilbiuose. Nukraujavimo atvejais 41 % apsvaigę nuo etanolio, kai AKK vidurkis 1,92‰±0,76. Šioje grupėje 59 % (n = 10) nustatytos pjautinės žaizdos kakle, yra kraujagyslių sužalojimų, 23 % (n = 4) rasta durtinių ir pjautinių žaizdų krūtinėje, kartu širdies ir kraujagyslių sužalojimai, 18 % buvo pjautinės žaizdos viršutinėse galūnėse, yra kraujagyslių sužalojimų. Amžiaus vidurkis, kai buvo nustatyta kaklo sužalojimų, – 61±20 metai, krūtinės sužalojimų – 39±12 metų, viršutinių galūnių sužalojimų – 71±9 metai. Visais atvejais nebuvo drabužių pažeidimų. Kaklo sužalojimo atvejais greta pagrindinės mirtį lėmusios žaizdos, bandomųjų, paviršinių sužalojimų skaičiaus vidurkis 8±4 (mediana 8), krūtinės sužalojimų atvejais – 5±2 (mediana 6); viršutinių galūnių sužalojimų atvejais – 6±1 (mediana 6). Bandomųjų žaizdų skaičius kakle, krūtinėje ir rankose statistiškai reikšmingai nesiskyrė (p > 0,05), tačiau buvo reikšmingai didesnis pasikorusiųjų grupėje (p < 0,05). Apsinuodijimo medikamentais grupėje nustatyta 16 bandomųjų pjūvių skaičiaus mediana, 42 % abiejuose dilbiuose. Statistiškai reikšmingo amžiaus skirtumo tarp apsinuodijimo medikamentais ir pasikorimo grupės nebuvo (p > 0,05), tačiau nukraujavimo atvejais skirtumas buvo statistiškai reikšmingas (p < 0,05). Nustatyta neigiama visos tiriamosios grupės amžiaus ir bandomųjų žaizdų skaičiaus koreliacija.

**Išvados:**

Savižudybės, padarytos duriančių ir pjaunančių savybių turinčiu daiktu, yra retas reiškinys, bei dažniausiai pasireiškia daugybiniais paviršiniais bandomaisiais sužalojimais, greta mirtį sukėlusios durtinės ir pjautinės žaizdos. Savižudybės aštriais daiktais dažniausios tarp vidutinio amžiaus vyrų bei daugiau nei pusė atvejų įvykdytos blaivių asmenų. Daugiausia bandomųjų pjūvių buvo matoma viršutinėse galūnėse, pasikorimo atvejais. Visais atvejais nebuvo matoma drabužių pažeidimų. Sužalojimų pobūdžio ir lokalizacijos vertinimas, tai, kad nėra gynybinių žaizdų ir drabužių pažeidimų, gali būti svarbus tiksliam vertinimui, ypač sudėtingais ir neaiškiais atvejais.

## Introduction

Suicide, i.e., the act of taking one’s own life, is a common cause of death worldwide. More than 720,000 people die by suicide each year. However, suicide by sharp objects is a relatively rare phenomenon, as it accounts only a few percent of all suicides. Sometimes, self-inflicted injuries can be mistaken for something else, such as an assault or an accident. The mortality rate from self-inflicted injuries with sharp objects is low; consequently, most suicide attempts do not lead to death. The low fatality rate associated with self-inflicted sharp force injuries may prompt the use of an additional method, which characterizes a complex suicide. Therefore, in this article, we will analyze the injuries caused by sharp objects in different suicide cases.

## Materials and Methods

### Study design and data source

In this retrospective study, we analyzed the injuries caused by sharp objects in case of complex suicide to determine correlations with suicide methods. From 2019 to 2024, nonconsecutive suicide autopsy cases where self-inflicted cuts were detected were selected from the data of impersonalized autopsies. All victims received full autopsies.

A search of scientific literature was conducted in the *PubMed* database by using the keywords ‘suicide’, ‘sharp object’, ‘stab wound’, ‘cut injuries’, and ‘incised wound’. The analysis focused on scientific literature that was published in last 10 years but also referred to older literature or scientific papers wherever strong arguments of high relevance were encountered.

### Identification of cases

The State Forensic Medicine Service (Lithuania) provided the autopsy data for 98 cases (71 men and 27 women). Cases involving self-inflicted cuts and stabs were specifically selected. Toxicological tests for alcohol and drugs were done for all cases. In every case, information was provided by the law enforcement agencies, including the possible crime location, time of death, and the presumed death mechanism.

### Limitations

The results cannot accurately reflect the condition of the entire global population. In our study, we only analyzed autopsy data.

### Statistical analysis

For statistical collected data processing, we used the *R Commander* and *Microsoft Excel* software. The Shapiro-Wilk test was used to determine whether the data were normally distributed. Spearman’s correlation coefficient was also assessed. R-values <0.39, 0.40–0.69, and ≥0.70 were defined as having weak, moderate, and strong correlations. Differences with *p* values less than 0.05 were considered significant.

### Toxicological analysis

Following the forensic dissection, blood and urine samples were collected for alcohol and drug testing. Headspace gas chromatography was used to detect the presence of alcohol, while liquid chromatography-time-of-flight mass spectrometry (LC/MS-TOF) and chromatography-tandem mass spectrometry (LC-MS/MS) were used for quantitative drug detection.

## Results

**Fig. 1:**
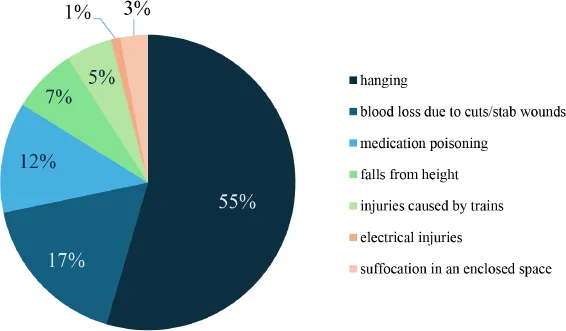
The main cause of death (in complex suicides) when the primary method of choice was unsuccessful

The mean age of the victims was 45.12 (± 17.93) years, with no statistically significant difference between the sexes (*p* = 0.97). In more than half of the cases (n = 52), suicides were committed while sober. Among the study group who were intoxicated with alcohol (n = 46), the mean blood alcohol concentration (BAC) was 1.96‰ ± 1.06, with no significant difference between the male and female groups (*p* = 0.73). The most common cause of death, where the primary method chosen (self-inflicted injuries by using sharp objects) failed, was hanging. This was followed by exsanguination and drug poisoning. (Fig. 1)

In cases of hanging, the mean age was 45 ± 15, and 60% were intoxicated (n = 32), with a mean BAC of 2.04‰ ± 1.08. A median of 13 attempted cuts was found, but, in all cases, the cuts were superficial (without damage to blood vessels). It is of importance to note that, in cases of hanging, a maximum number of 88 superficial cuts on various parts of the body were observed in one victim. The most common site of injury was the forearms, with injuries more frequently found on the left forearm (n = 43), but as many as 26% were found on both the right and left forearms. Injuries (superficial cuts) were also found in other parts of the body, such as both legs and the neck.

When the primary cause of death was exsanguination due to cut/stab wounds, 41% were intoxicated with alcohol (mean BAC was 1.92‰ ± 0.76). In this group, 59% (n = 10) had cut neck wounds with damage to the neck blood vessels; 23% (n = 4) had stab wounds to the chest with damage to the heart and major blood vessels, and 18% (n = 3) had cuts to the upper limbs with arterial injury. It can be concluded that injuries to the extremities rarely result in death. When cut wounds to the neck were determined to be the cause of death in suicide cases, the mean age was 61 ± 20 years, whereas, in cases of stab/cut wounds to the chest, it was 39 ± 12 years, and in cases of cut wounds to the upper limbs, it was 71 ± 9 years. In all cases, no clothing damage was found. In cases of neck injuries, in addition to the main wound that caused death, a mean of 8 ± 4 (median 8) superficial neck cuts were found near the main injury. A mean of 5 ± 2 (median 6) and 6 ± 1 (median 6) superficial trial injuries was observed in cases related to chest and upper limbs, respectively. The number of trial wounds on the neck, chest, and arms did not differ considerably (*p* >0.05), but deviated statistically significantly from the number of trial wounds in the hanging group (*p* <0.05), there were usually more trial incisions.

Concerning the third most frequent cause of death, medication poisoning, the mean age was 35.5 ± 12.5 years, and the trial cuts were superficial in all cases (without damage to blood vessels). Median of 16 trial cuts were found, 42% of which were on both forearms. There was no statistically significant difference in the mean age between cases of toxic drug exposure and hanging (p > 0.05), but there was a significant difference when the cause of death was exsanguination after cuts/stab wounds (p < 0.05) (Table 1).

**Table 1:** Overview of complex suicide cases in the present study

Main cause of death	Mean age (years)	Hesitation (trial) wounds location	Hesitation wounds number (median)
*Hanging (n=54)*	45.12 ± 17.93	Forearms (mostly on the left side) Legs Neck	13
*Exsanguination (n=17):*			
➢ *cut injuries to the neck (n=10)*	61 ± 20	Near the main injury	8
➢ *stab/cut injuries to the chest (n=4)*	39 ± 12	Near the main injury	6
➢ *cut injuries to the upper limbs (n=3)*	71 ± 9	Near the main injury	6
*Medication poisoning (n=12)*	35.5 ± 12.5	Forearms	16

The Spearman’s correlation test was used to evaluate the strength of the correlation between the age of the entire study group (n = 98) and the number of trial wounds, a statistically significant weak negative correlation was observed (r = -0.33, *p* <0.05). This indicates that, as the age of the victims increases, the number of trial incisions decreases.

## Discussion

Suicide, defined as the intentional termination of one’s own life, is a leading cause of death worldwide. Over 720,000 individuals take their own lives each year [1]. In general, suicide by sharp objects is a relatively rare phenomenon – specifically, in Western countries and around the world, it accounts about 3% of all suicides [2,3]. The reason is that the mortality rate from self-harm with sharp objects is low, and therefore most suicide attempts end in survival. However, this is a clinically significant problem because of the risk of permanent disability and repeated suicide attempts [4,5]. Several studies have found that a previous suicide attempt is the strongest risk factor for death by suicide, followed by mental illness and alcohol abuse [6]. From a classical perspective, injuries caused by sharp objects are classified into three types: cuts, stab wounds, and lacerations [7]. From an anatomical point of view, cut wounds can be considered horizontal injuries (when the length of the wound is greater than its depth), while the less common stab wounds are vertical injuries (the depth of the wound usually exceeds its length in the skin). The most common tool used in suicide attempts was a knife, followed by glass [5,7,8]. It has been observed that suicides involving sharp objects are typically committed by victims who have previously been diagnosed with a psychiatric disorder and/or have already attempted to take their own lives (as evidenced by old scars or cuts made during the same suicide attempt) [9,10]. People with psychiatric disorders tend to have more severe wounds, as well as stab wounds in unusual places (bladder, eyes) or unusual sharp objects, such as their own teeth, dentures, electric saws, scissors, etc. [11–13]. In most fatal cases, victims have multiple wounds (bilateral wrist injuries, multiple stab wounds), i.e., it is not common for the death to occur after a single stab or cut wound. The fact that some of the victims had also made more than five separate cuts confirms the seriousness of their intentions [14,15].

### Cutting wounds

Historically, the act of taking one’s own life using sharp objects was considered a ritual form of suicide. The rituals of *seppuku* or *hara-kiri* are well known in Japan. Whereas, in the Celtic culture, voluntary death by cutting one’s throat was considered a form of celebration of life. To this day, many bloody rituals involving cuts and stab wounds made with sharp objects are practiced by the Masai tribe in Africa and some Aboriginal communities in Australia [3]. In developed countries, cuts are most often associated with self-harm, whether or not the intention is to take one’s own life, and are found on easily accessible parts of the body, such as the head, neck, and hands. Fatal cuts are most often found on the hands and neck [7]. Wrist injuries are not uncommon in forensic medicine and can range from simple skin lacerations to deep injuries. This severely damages anatomical structures such as arteries, tendons, and nerves, as well as their functions [5].

### Stabbing wounds

Self-harm by stabbing with sharp objects is a rare method of suicide, accounting for about 1% of suicide attempts [16]. Many autopsy studies have shown that people who commit suicide by stabbing themselves with a sharp instrument choose easily accessible locations, i.e., the upper limbs, neck, and parts of the body containing vital organs (abdomen, heart area), the damage to which results in a quick and relatively painless death [3,17]. Since most knife wounds resulting in suicide were found on the left side of the front of the chest, it can be assumed that the intention was to injure the heart and thus achieve a fatal outcome [2]. Stabs to the abdomen can be considered attempts to stab the heart, as they were mostly made in the upper abdomen, and their trajectories were mostly directed upwards, thus reaching the chest [18]. Factors such as indecisiveness or less forceful stabbing could explain the rarity of blood vessel injuries in suicides, as the large blood vessels are deep within the torso and difficult to reach with superficial stabs to the front of the torso. The small number of suicides involving stab wounds that damaged blood vessels, the heart or blood vessels near the heart (the thoracic aorta, the coronary artery, and the pulmonary artery) can be explained by the fact that the victims were attempting to stab themselves in the heart [2].

### Complex suicides

Quite often, wounds caused by sharp objects occur in cases of complex suicides. Forensic medicine literature indicates that complex suicides account for up to 5.0% of all suicides [10]. A complex suicide is defined as the use of more than one method to ensure death by suicide [19]. It should be noted that, in cases of unplanned complex suicides, the primary method of suicide is sharp force injuries (especially wrist cuts). However, this method is usually not sufficient, and thus it consequently gets abandoned in favor of another one (hanging, drowning, jumping from a height, etc.) [8,12,19].

### Differences between wounds caused by sharp objects in suicides and homicides

In most cases, injuries caused by sharp objects are associated with homicides rather than suicides. The ratio is approximately 1:5 (suicides:homicides). The most important factor in differentiating between suicide and homicide deaths is the absence of defensive wounds or the presence of trial cuts [17]. The most common locations for defensive wounds are the fingers, palms, and back of the hand, predominantly on the left hand and the palm of the right hand [20]. Hesitation or trial wounds are found in about 70% of suicide cases and are usually superficial, multiple, and parallel to the main injury. More information is provided by the orientation of the stab wound, which is usually horizontal if self-inflicted and vertical if inflicted by another person [8,15]. In right-handed individuals, self-inflicted injuries are usually on the opposite side of the body, running from left to right [3,21]. The absence of clothing damage may also be important [21]. In our paper, we can also observe these patterns.

## Conclusions

Suicides by sharp objects are a relatively rare phenomenon; they most commonly involve multiple superficial trial injuries alongside a fatal stab or incised wound. Suicides with sharp objects are most common among middle-aged men, and, in more than half of the cases, the individuals were sober at the time of the event. The majority of trial cuts were observed on the upper limbs, especially in cases of hanging. In all instances, no damage to clothing was observed. Recognition of the injury patterns and locations, along with the absence of defensive wounds and clothing damage, could be essential for accurate forensic interpretation, particularly in complex and ambiguous cases.
